# *m*-Iodosylbenzoic acid – a convenient recyclable reagent for highly efficient aromatic iodinations

**DOI:** 10.1186/1860-5397-3-19

**Published:** 2007-06-04

**Authors:** Andreas Kirschning, Mekhman S Yusubov, Roza Y Yusubova, Ki-Whan Chi, Joo Y Park

**Affiliations:** 1 Institut für Organische Chemie and Zentrum für Biomolekulare Wirkstoffchemie (BMWZ), Leibniz Universität Hannover, Schneiderberg 1B, D-30167 Hannover, Germany; 2 The Siberian State Medical University, 2 Moskovsky trakt, 634050 Tomsk, Russia and The Tomsk Polytechnic University, 30 Lenin st., 634050 Tomsk, Russia; 3 University of Ulsan, 680-749 Ulsan, Republic of Korea

## Abstract

*m*-Iodosylbenzoic acid performs iodinations of arenes in the presence of iodine at room temperature in acetonitrile. Separation of pure products is conveniently achieved by scavenging any aryl iodide by ion exchange with IRA-900 (hydroxide form). The reduced form of the reagent, *m*-iodobenzoic acid, can be easily recovered from the ion exchange resin or from the basic aqueous solution by simple acidification with HCl.

## Background

**Figure 1:**
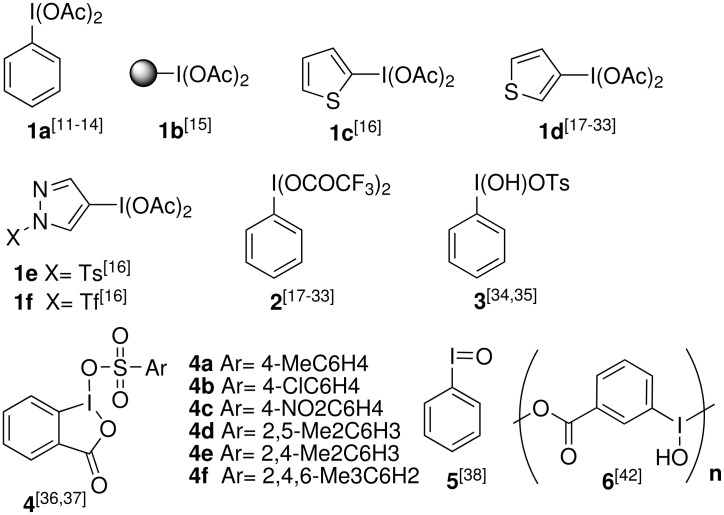
Hypervalent iodine reagents **1** – **6**.

In recent years, iodoarenes have gained increasing importance because they are widely used as building blocks in organic synthesis. They are particularly important as indispensable substrates for numerous methods of N-N bond formation, [1–2] for the chemistry of heterocyclic [3] and organometallic compounds, [4–8] and for the synthesis of polyvalent iodine organic compounds. [9–10] In addition, polyvalent organoiodine compounds have served as cooxidants in the iodination of arenes. [11–36] Typical polyvalent iodine sources for these iodination reactions are reagents **1**–**4** (Figure 1). Iodosylbenzene **5** is not suitable for iodinations because of its low activity. [37]

In this report we describe a practical improvement for these iodinations as far as purification of the products and recycling of the iodine reagent is concerned. The broad use of hypervalent iodine reagents is still hampered by tedious purification and recycling protocols. Commonly, purification relies on chromatography. Recently, tagging strategies for reagents and catalysts have widely been investigated that allow easy purification by means of specific phase separation or scavenging. [39–41]

## Results and discussion

**Scheme 1:**
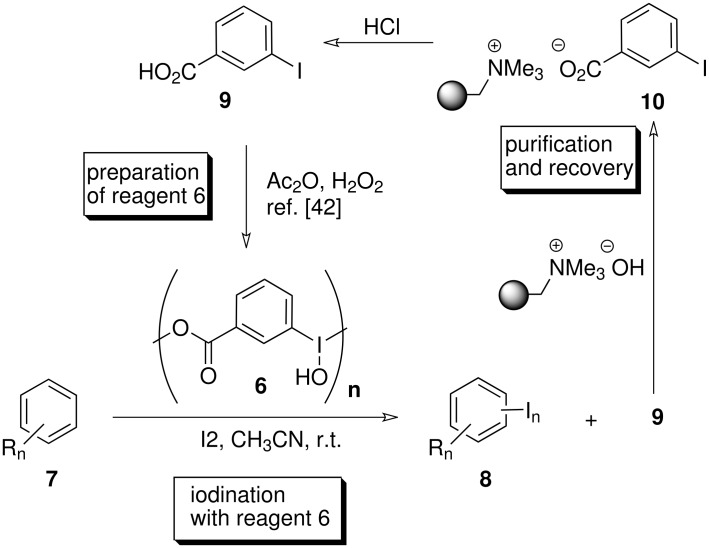
Iodine(III)-promoted iodination of arenes and concept of purification.

In this context, we recently described an improved procedure for the preparation of the hardly known *m*-iodosylbenzoic acid **6** and showed that it is a recyclable reagent for the highly efficient RuCl_3_-catalyzed oxidation of alcohols to aldehydes and ketones. [42] In the present work we demonstrate the utility of *m*-iodosylbenzoic acid **6** as a recyclable reagent for the iodination of arenes. In fact reagent **6** can be regarded as a tagged version of iodoso benzene **5** which, if used in access, can be conveniently removed at the end of the reaction by filtration after addition of IRA 900 (hydroxide form) (Scheme 1). This scavenging concept can also be applied to reduction products such as *m*-iodobenzoic acid **9**. Importantly, **9** which also serves as the starting material for the preparation of **6** can easily be regenerated (> 95%) from polymer **10** in pure form by treatment with aqueous HCl.

We found that the reaction of aromatic substrates **7a-o** with I_2_ and **6** in CH_3_CN (commonly in the presence of 50% aqueous H_2_SO_4_) led to the corresponding iodinated arenes in 40 – 99% yield under mild conditions (Scheme 1 and Table 1 and Table 2). Addition of aqueous H_2_SO_4_ accelerated the iodination of benzenes. For heteroarenes **7j** and **7o** this additive was not required and if an additional alcohol group was present (see **7n**), addition of aqueous H_2_SO_4_ resulted in its oxidation. Compared to diacetoxyiodobenzene (DIB) **1a** and its polymeric analog **1b**, the use of *m*-iodosylbenzoic acid **6** for mono- and diiodination requires the use of smaller amounts of iodine as well as of the polyvalent iodine reagent. [15] For example, the preparation of 2,4-diiodoanisole **8k** from anisole **7k** in the presence of **1b** was achieved using 4.8 equiv. of both iodine and **1b** while our iodination protocol required only 2.4 equiv. of iodine and 1.2 equiv. of *m*-iodosylbenzoic acid **6**.

**Table 1:** Monoiodination of arenes with *m*-iodosylbenzoic acid **6** (see Supporting Information File 1 for full experimental data).

Arene	Iodoarene	Conditions	Yield (%)^a^	mp or bp °C (lit. mp)

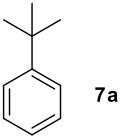	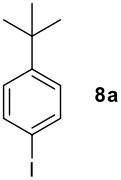	5 h, 60°C	91	250–254 (249 – 254; [43])
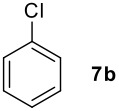	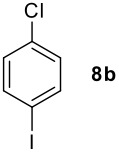	24 h, rt	76^b^	Determined by GC-analysis
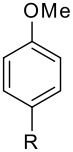	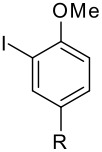			
**7c** R = Br	**8c** R = Br	0.5 h, rt	92	**8c** 62–64 (oil; [10])
**7d** R = -C(O)Ph	**8d** R = -C(O)Ph	0.2 h, rt^c^	90	**8d;** ref. 44) 70–72 (71–72[44])
**7e** R = -C(O)CH_3_	**8e** R = -C(O)CH_3_	0.1 h, rt^c^	90	**8e** 101–103 (103.6; [45])
**7f** R = -CH_2_C(O)CH_3_	**8f** R = -CH_2_C(O)CH_3_	0.1 h, rt^c^	79	**8f** oil (oil; [46])
**7g** R = -CHO	**8g** R = -CHO	2.0, rt^c^	85	**8g** 103–105 (105–10; [47])
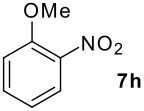	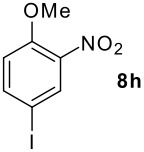	16 h, rt	40	95–96 (96; [48])
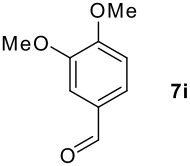	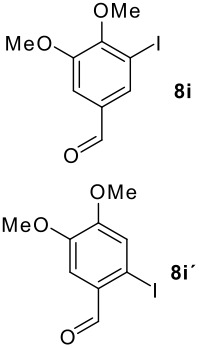	3.0 h, rt	60	**8i : 8i'** = 1.0 : 0.8
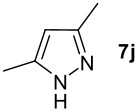	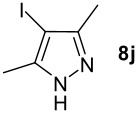	1.0 h, rt^d^	97^e^	134–135 (134–136; [49])

^a^ Molar ratio ArH/**6**/iodine 0.2/0.24/0.12 (in mmol) and 0.05 mL aq. (50%) H_2_SO_4_; isolated yields. ^b^ Determined by GC-analysis. ^c^ Instead of 0.05 mL only 0.02 mL aq. (50%) H_2_SO_4_ was added. ^d^ No aq. (50%) H_2_SO_4_ was added. ^e^NaHCO_3_ was used instead of IRA 900 (hydroxide form).

**Table 2:** Diiodination of Arenes with *m*-Iodosylbenzoic acid **6** (see Supporting Information File 1 for full experimental data).

Arene	Diiodoarene	Conditions	Yield (%)^a,b^	mp, °C (lit. mp)

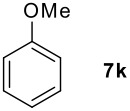	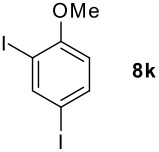	5 h, rt	91	66–67 (67.5–68.5; [10])
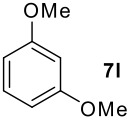	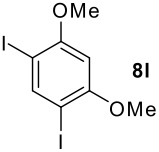	1.0, rt	99	198–199 (199–200; [50])
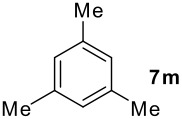	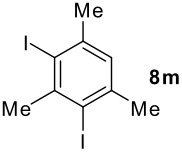	0.2 h, rt	96	80–81 (79–80 [10])
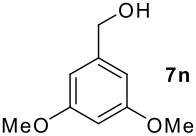	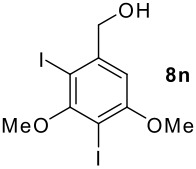	2 h, rt^c^	83	146.5–147.5°C (decomp.)
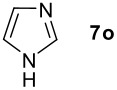	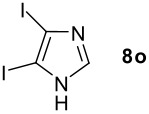	1 h, rt^c^	74^d^	189–191 (191–192; [51])

^a^ Isolated yields. ^b^ Molar ratio ArH/**6**/iodine 0.2/0.48/0.24 (in mmol) and 0.05 mL aq. (50%) H_2_SO_4_. ^c^ No aq. (50%) H_2_SO_4_ was added. ^d^ NaHCO_3_ was used instead of IRA 900 (hydroxide form).

Likewise, for the preparation of aryliodide **8c** a 2.4 molar excess of both iodine and reagent **1a** had to be employed while in our case 1.2 equiv. of iodine and 1.2 equiv. of reagent **6** were required for full conversion.

As is evident from the tables, iodination of arenes that are acylated like **7d**,**e**,**g** and **7i** commonly led to excellent yields of selectively iodinated arenes **8d**,**e**,**g** and **8i**. Increasing the nucleophilicity of the aromatic ring such as in 3,5-dimethoxybenzyl alcohol **7n** led to diiodinated benzyl alcohol **8n** in good yield. Oxidation of the alcohol group was not observed.

**Scheme 2:**
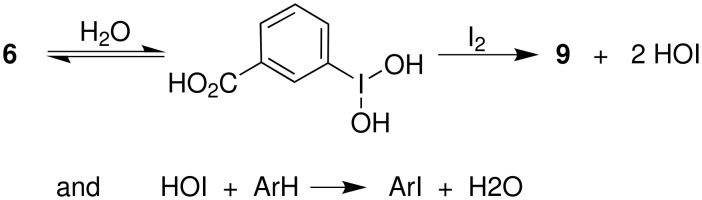
Proposed intermediates.

Based on related iodine(III)-mediated iodinations of arenas [12–37] we suggest that the hydrated form of **6** oxidizes iodine to HOI which serves as the reactive electrophilic intermediate (Scheme 2).

From the results collected it can be concluded that *m*-iodosylbenzoic acid **6** shows a similar reactivity as 1-(arenesulfonyloxy)benziodoxolones **4a-f** [36–37]. However, reagent **6** is cheaper and exerts better selectivity in the iodination reactions.

## Conclusion

In conclusion, we disclose that the rarely employed *m*-iodosylbenzoic acid is an ideal tagged iodine(III) reagent which in our view allows the easiest purification protocol for aryliodine reagents known so far. This tagging concept was utilized in the mild iodination of arenes but could potentially be applied to most other iodine(III)-mediated reactions.

## Supporting Information

File 1 Experimental details. The data provide general experimental details as well as an improved procedure for the preparation of m-iodosylbenzoic acid (**6**), a typical iodination procedure and spectroscopic and analytic data for **8n**.
