# Supplementary material for: m-Iodosylbenzoic acid – a convenient recyclable reagent for highly efficient aromatic iodinations
Source: Beilstein J Org Chem. 2007 Jun 4;3:19. doi: 10.1186/1860-5397-3-19 (PMC1924527; doi:10.1186/1860-5397-3-19)
Supplement: File 1 — Experimental details. The data provide general experimental details as well as an improved procedure for the preparation of m-iodosylbenzoic acid (6), a typical iodination procedure and spectroscopic and analytic data for 8n. [file Beilstein_J_Org_Chem-03-19-s001.doc]

**Additional file 1**

NMR spectra were recorded with a Bruker DPX-400 spectrometer at 400 MHz (^1^H NMR) and at 100 MHz (^13^C NMR) in CDCl_3_ using tetramethylsilane as the internal standard. Mass spectra (EI) were obtained at 70 eV with a type VG Autospec apparatus (Micromass). GC analyses were conducted using a Hewlett-Packard HP 6890 Series GC System equipped with a SE-54 capillary column (25 m, Macherey-Nagel) and a FID detector 19231 D/E. IR spectra were recorded with a Brucker FT-IR Vector 22 apparatus. Melting points were determined in open glass capillaries with a Gellenkamp apparatus and are uncorrected. Analytical thin-layer chromatography was performed using precoated silica gel 60 F_254_ plates (Merck, Darmstadt), and the spots were visualised with UV light at 254 nm or by staining with H_2_SO_4_/4-methoxybenzaldehyde in ethanol. Flash column chromatography was performed on Merck silica gel 60 (230-400 mesh). Commercially available reagents and dry solvents (acetonitrile) were used as received. Most compounds prepared were isolated after filtration and removal of the solvent. Pure homogeneous product samples were collected after flash chromatography and purity was analyzed by TLC, GC and NMR analyses. Characterization was achieved by appropriate spectroscopic methods (^1^H and ^13^C NMR, IR, high resolution MS) and comparison with literature data.

*Improved procedure for the preparation of m-iodosylbenzoic acid (****6****)*

*m*-Iodobenzoic acid **9** (2.48 g, 10 mmol) was added at 40 ^o^C to a freshly prepared solution of peracetic acid in acetic acid [prepared by stirring a mixture of acetic anhydride (60 mL) and 35% H_2_O_2_ (14 mL) at 40 ^o^C for 4 h in dark] and the resulting mixture was stirred for 12 h at ambient temperature in dark. A spontaneous heating of the reaction mixture up to 45 ^o^C and complete dissolution of *m*-iodobenzoic acid was observed during the first two hours of stirring. The reaction mixture was poured onto 150 mL of ice-water, and allowed to crystallize. The precipitate was filtered on a Büchner funnel and washed with another 200 mL of ice-water. The solid was then dried, first by maintaining suction and then in vacuum, to afford product **2** as a light yellow powder. Yield 1.9-2.11 g (72-80% yield); m.p. 168-169 °C. ^1^H NMR (200 MHz, CD_3_COOD): δ = 7.71 (t, 1H), 8.35 (d, 1H), 8.48 (d, 1H), 8.89 (s, 1H). Anal. Calcd for C_7_H_5_IO_5_ (263.93): C, 31.84; H, 1.91. Found: C, 31.77; H, 1.84.

*Typical iodination procedure:*

To a mixture of arenes **7a-j** (0.2 mmol), iodine (0.12 mmol) and *m*-iodosylbenzoic acid **6** (0.24 mmol) in MeCN (0.5 mL) was added aq. H_2_SO_4_ (50%, 0.5 mL) and the reaction mixture was stirred at room temperature as indicated in the tables (the reactions were monitored by TLC). Then, dichloromethane (1.5 mL) and IRA 900 (700-750 mg; hydroxide form) were added and the mixture was shaken for 5 min. The polymer was removed by filtration and the solution was concentrated in vacuo to afford pure iodoarenes **8a-j** as judged by NMR-spectroscopy. *m*-Iodobenzoic acid **9** can easily be regenerated from IRA 900 resin **10** by treatment with aqueous HCl and reoxidized to reagent **6** without additional purification as described in reference [42]. For diiodination the same protocol was applied except that the molar amounts of reagent **6** and iodine were doubled.

*Spectroscopic and analytic data for* ***8n***:

IR (KBr): ν = 3324 (OH), 1150, 1043 (C-O-), 728 (C-I) cm^–1^; ^1^H NMR (400 MHz, CD_3_OD) δ = 3.91 (s, 6H, CH_3_O), 5.15 (s, 2H, CH_2_), 6.60 ppm (s, 1H_arom._); ^13^C NMR (400 MHz, CD_3_OD) δ = 57.27 (q, CH_3_O), 74.85 (t, CH_2_OH), 82.49 (s, CI), 96.06 (d, C_arom._), 146.08 (s, *C*_arom._-CH_2_), 161.19 ppm (s, *C*_arom._-OCH_3_).

HRMS (TOF ES): calculated for [M-H] C_9_H_9_I_2_O_3_: 418.8647; found : 418.8635
